# Supplementary material for: A location-based service scheme with attribute information privacy
Source: PLoS One. 2024 Sep 6;19(9):e0309919. doi: 10.1371/journal.pone.0309919 (PMC11379283; doi:10.1371/journal.pone.0309919)
Supplement: S1 Data set — (ZIP) [file pone.0309919.s001.zip › Data sets/Geolife Trajectories 1.3/User Guide-1.3.pdf]

User Guide

Version 1.3 (2012/08/01)

1. Data Description

This GPS trajectory dataset was collected in (Microsoft Research Asia) Geolife project by 182 users in a period of over five years (from April 2007 to August 2012). A GPS trajectory of this dataset is represented by a sequence of time-stamped points, each of which contains the information of latitude, longitude and altitude. This dataset contains 17,621 trajectories with a total distance of 1,292,951kilometers and a total duration of 50,176 hours. These trajectories were recorded by different GPS loggers and GPS-phones, and have a variety of sampling rates. 91.5 percent of the trajectories are logged in a dense representation, e.g. every 1~5 seconds or every 5~10 meters per point.

This dataset recoded a broad range of users' outdoor movements, including not only life routines like go home and go to work but also some entertainments and sports activities, such as shopping, sightseeing, dining, hiking, and cycling. This trajectory dataset can be used in many research fields, such as mobility pattern mining, user activity recognition, location-based social networks, location privacy, and location recommendation.

Although this dataset is wildly distributed in over 30 cities of China and even in some cities located in the USA and Europe, the majority of the data was created in Beijing, China. Figure 1 plots the distribution (heat map) of this dataset in Beijing. The figures standing on the right side of the heat bar denote the number of points generated in a location.

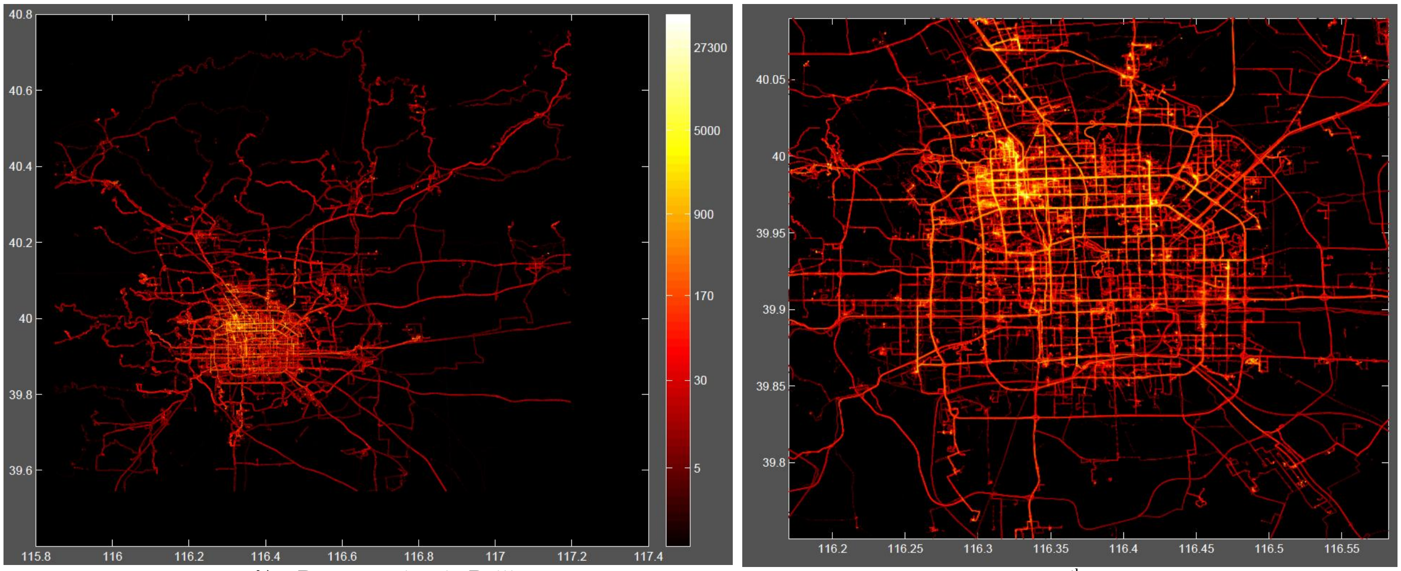

A) Data overview in Beijing

B) Within the 5th Ring Road of Beijing

Figure 1 Distribution of the dataset in Beijing city

The distributions of distance and duration of the trajectories are presented in Figure 2 and Figure 3.

In the data collection program, a portion of users have carried a GPS logger for years, while some of the others only have a trajectory dataset of a few weeks. This distribution is presented in Figure 4, and the distribution of the number of trajectories collected by each user is shown in Figure 5.

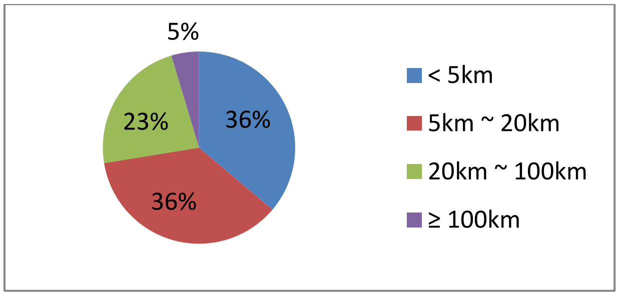

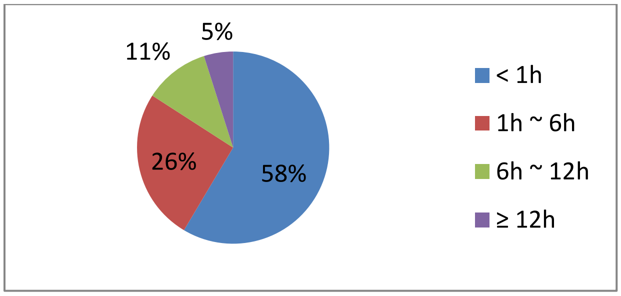

Figure 2 Distribution of trajectories by distance

Figure 3 Distribution of trajectories by effective duration

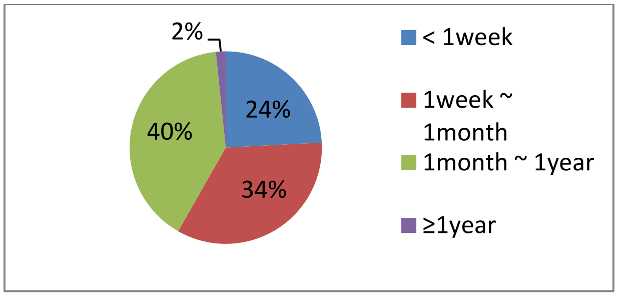

Figure 4 Distribution of users by data collection period

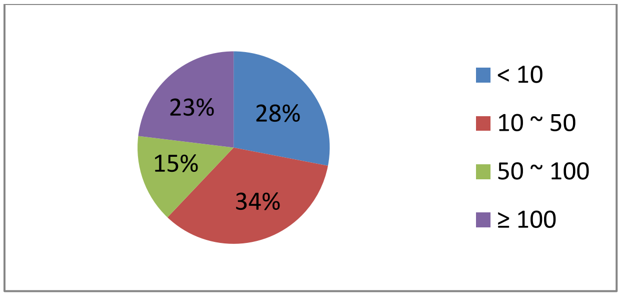

Figure 5 Distribution of users by trajectories

2. What's new?

2.1. Transportation mode labels

73 users have labeled their trajectories with **transportation mode**, such as driving, taking a bus, riding a bike and walking. There is a label file storing the transportation mode labels in each user's folder. See section 4.2 for the format of labels.

The total distance and duration of transportation modes are listed in Figure 6. Though this only covers a part of the dataset used in the following papers, the scale of this released dataset can still support transportation mode learning.

Transportation mode	Distance (km)	Duration (hour)
Walk	10,123	5,460
Bike	6,495	2,410
Bus	20,281	1,507
Car & taxi	32,866	2,384
Train	36,253	745
Airplane	24,789	40
Other	9,493	404
Total	14,0304	12,953

Figure 6 Total distance and duration of transportation modes

2.2. Changes on the scale of dataset

Changes on the scale of dataset between version 1.2 and version 1.3 are listed in Figure 7. Effective days refer to the total number of days where there's a record in the dataset.

	**Version 1.2**	**Version 1.3**	**Change**
Time span of the collection	04/2007 – 10/2011	04/2007 – 8/2012	+10 months
Number of users	178	182	+4
Number of trajectories	17,621	18,670	+1,049
Number of points	23,667,828	24,876,978	+1,209,150
Total distance	1,251,654km	1,292,951km	+41,297 km
Total duration	48,203hour	50,176hour	+1,973 hour
Effective days	10,413	11,129	+716

Figure 7 Changes on the scale of dataset

3. Paper Citation

Please cite the following papers when using this GPS dataset.

[1] Yu Zheng, Lizhu Zhang, Xing Xie, Wei-Ying Ma. Mining interesting locations and travel sequences from GPS trajectories. In Proceedings of International conference on World Wild Web (WWW 2009), Madrid Spain. ACM Press: 791-800.

[2] Yu Zheng, Quannan Li, Yukun Chen, Xing Xie, Wei-Ying Ma. Understanding Mobility Based on GPS Data. In Proceedings of ACM conference on Ubiquitous Computing (UbiComp 2008), Seoul, Korea. ACM Press: 312-321.

[3] Yu Zheng, Xing Xie, Wei-Ying Ma, GeoLife: A Collaborative Social Networking Service among User, location and trajectory. Invited paper, in IEEE Data Engineering Bulletin. 33, 2, 2010, pp. 32-40.

4. Data Format

4.1. Trajectory file

Every single folder of this dataset stores a user's GPS log files, which were converted to PLT format. Each PLT file contains a single trajectory and is named by its starting time. To avoid potential confusion of time zone, we use **GMT** in the date/time property of each point, which is different from our previous release.

PLT format:

Line 1…6 are useless in this dataset, and can be ignored. Points are described in following lines, one for each line.

Field 1: Latitude in decimal degrees.

Field 2: Longitude in decimal degrees.

Field 3: All set to 0 for this dataset.

Field 4: Altitude in feet (-777 if not valid).

Field 5: Date - number of days (with fractional part) that have passed since 12/30/1899.

Field 6: Date as a string.

Field 7: Time as a string.

Note that field 5 and field 6&7 represent the same date/time in this dataset. You may use either of them.

Example:

39.906631,116.385564,0,492,40097.5864583333,2009-10-11,14:04:30

39.906554,116.385625,0,492,40097.5865162037,2009-10-11,14:04:35

4.2. Transportation mode labels

Possible transportation modes are: *walk*, *bike*, *bus*, *car*, subway, *train*, *airplane*, *boat*, *run* and *motorcycle*. Again, we have converted the date/time of all labels to **GMT**, even though most of them were created in China.

Example:

Start Time	End Time	Transportation Mode
2008/04/02 11:24:21	2008/04/02 11:50:45	bus
2008/04/03 01:07:03	2008/04/03 11:31:55	train
2008/04/03 11:32:24	2008/04/03 11:46:14	walk
2008/04/03 11:47:14	2008/04/03 11:55:07	car

First, you can regard the label of both *taxi* and *car* as *driving* although we set them with different labels for future usage. Second, a user could label the transportation mode of a *light rail* as *train* while others may use *subway* as the label. Actually, no trajectory can be recorded in an underground subway system since a GPS logger cannot receive any signal there. In Beijing, the light rails and subway systems are seamlessly connected, e.g., line 13 (a light rail) is connected with line 10 and line 2, which are subway systems. Sometimes, a line (like line 5) is comprised of partial subways and partial light rails. So, users may have a variety of understanding in their transportation modes. You can differentiate the real train trajectories (connecting two cities) from the light rail trajectory (generating in a city) according to their distances. Or, just treat them the same.

5. Contact

If you have any questions about this dataset, please contact Dr. Yu Zheng from Microsoft Research Asia.

Yu Zheng

Tel: 86-10-59173038   Email: yuzheng@microsoft.com

Homepage: http://research.microsoft.com/en-us/people/yuzheng/default.aspx

Address: Microsoft Research Asia, Tower 2, No. 5 Danling Street, Haidian District, Beijing, P.R. China 100080

6. Release history

Version 1.3 (released 2012/08/01)

Version 1.2 (released 2011/10/31)

Version 1.1 (released 2011/07/25)

Version 1.0 (released 2010/09/30)

7. Microsoft Research License Agreement

Non-Commercial Use Only

<< GeoLife GPS Trajectories>>

This Microsoft Research License Agreement, including all exhibits ("MSR-LA") is a legal agreement between you and Microsoft Corporation (Microsoft or we) for the software or data identified above, which may include source code, and any associated materials, text or speech files, associated media and "online" or electronic documentation and any updates we provide in our discretion (together, the "Software").

By installing, copying, or otherwise using this Software, you agree to be bound by the terms of this MSR-LA. If you do not agree, do not install copy or use the Software. The Software is protected by copyright and other intellectual property laws and is licensed, not sold.

SCOPE OF RIGHTS:

You may use this Software for any non-commercial purpose, subject to the restrictions in this MSR-LA. Some purposes which can be non-commercial are teaching, academic research, public demonstrations and personal experimentation. You may not distribute this Software or any derivative works in any form. In return, we simply require that you agree:

1. That you will not remove any copyright or other notices from the Software.

2. That if any of the Software is in binary format, you will not attempt to modify such portions of the Software, or to reverse engineer or decompile them, except and only to the extent authorized by applicable law.

3. That Microsoft is granted back, without any restrictions or limitations, a non-exclusive, perpetual, irrevocable, royalty-free, assignable and sub-licensable license, to reproduce, publicly perform or display, install, use, modify, post, distribute, make and have made, sell and transfer your modifications to and/or derivative works of the Software source code or data, for any purpose.

4. That any feedback about the Software provided by you to us is voluntarily given, and Microsoft shall be free to use the feedback as it sees fit without obligation or restriction of any kind, even if the feedback is designated by you as confidential.

5. THAT THE SOFTWARE COMES "AS IS", WITH NO WARRANTIES. THIS MEANS NO EXPRESS, IMPLIED OR STATUTORY WARRANTY, INCLUDING WITHOUT LIMITATION, WARRANTIES OF MERCHANTABILITY OR FITNESS FOR A PARTICULAR PURPOSE, ANY WARRANTY AGAINST INTERFERENCE WITH YOUR ENJOYMENT OF THE SOFTWARE OR ANY WARRANTY OF TITLE OR NON-INFRINGEMENT. THERE IS NO WARRANTY THAT THIS SOFTWARE WILL FULFILL ANY OF YOUR PARTICULAR PURPOSES OR NEEDS.

6. THAT NEITHER MICROSOFT NOR ANY CONTRIBUTOR TO THE SOFTWARE WILL BE LIABLE FOR ANY DAMAGES RELATED TO THE SOFTWARE OR THIS MSR-LA, INCLUDING DIRECT, INDIRECT, SPECIAL, CONSEQUENTIAL OR INCIDENTAL DAMAGES, TO THE MAXIMUM EXTENT THE LAW PERMITS, NO MATTER WHAT LEGAL THEORY IT IS BASED ON.

7. That we have no duty of reasonable care or lack of negligence, and we are not obligated to (and will not) provide technical support for the Software.

8. That if you breach this MSR-LA or if you sue anyone over patents that you think may apply to or read on the Software or anyone's use of the Software, this MSR-LA (and your license and rights obtained herein) terminate automatically. Upon any such termination, you shall destroy all of your copies of the Software immediately. Sections 3, 4, 5, 6, 7, 8, 11 and 12 of this MSR-LA shall survive any termination of this MSR-LA.

9. That the patent rights, if any, granted to you in this MSR-LA only apply to the Software, not to any derivative works you make.

10. That the Software may be subject to U.S. export jurisdiction at the time it is licensed to you, and it may be subject to additional export or import laws in other places. You agree to comply with all such laws and regulations that may apply to the Software after delivery of the software to you.

11. That all rights not expressly granted to you in this MSR-LA are reserved.

12. That this MSR-LA shall be construed and controlled by the laws of the State of Washington, USA, without regard to conflicts of law. If any provision of this MSR-LA shall be deemed unenforceable or contrary to law, the rest of this MSR-LA shall remain in full effect and interpreted in an enforceable manner that most nearly captures the intent of the original language.

Copyright (c) Microsoft Corporation. All rights reserved.
