# Supplementary material for: A location-based service scheme with attribute information privacy
Source: PLoS One. 2024 Sep 6;19(9):e0309919. doi: 10.1371/journal.pone.0309919 (PMC11379283; doi:10.1371/journal.pone.0309919)
Supplement: Attachment — Submitted filename: Response to Reviewers.docx [file pone.0309919.s002.docx]

Reviewers' comments:

**Reviewer #1:** In this paper, the authors try to propose a CP-ABE based PIR by utilizing CP-ABE technology.
However, the following questions should be considered.
(1) Inadequate motivation and problem formulation: The introduction fails to convincingly motivate the need for a CP-ABE based PIR. The specific research problems and challenges that the paper aims to address are not clearly articulated. The claimed contributions are not well-positioned against prior works, making it hard to gauge their significance. A stronger case needs to be made for why the proposed CP-ABE based PIR is an important advancement over existing solutions.

(2) Lack of technical depth and insights: The proposed construction does not offer significant technical innovations beyond combining existing techniques from CP-ABE based PIR. The paper lacks new concepts, insights, or design principles that push forward the state-of-the-art in this domain.

(3) In this paper, the authors proposed a CP-ABE based PIR by utilizing CP-ABE technology. However, the authors do not give the related works about CP-ABE.

(4) The authors proposed a CP-ABE based PIR. However, they do not give the concrete name of the related algorithms.

(5) The proposed CP-ABE based PIR is not complete. Especially, key generation algorithm is not given. How can you encrypt and decrypt?

**Reviewer #2:**

1. It is not clear, what is the role of f(x) in algorithm 1?
2. No threat model is presented in the paper. It will be good to include the threat model and present the security analysis accordingly.

3. It is not clear whether the LBS will know who is sending the query or not? If the LBS knows the individual who sends the query then privacy is lost.

4. Decryption proof of correctness may be with all steps involved. It is not clear how the second step is derived from the first step.

5. Zero Knowledge conceal is used in table 2 however no where it is described.

6. Figure 8 should be rechecked. X-axis is not clear. Is it time or the number of queries? Similarly figure 6.

7. The novelty should be clearly presented.

**Reviewer #3:** In this manuscript, in this paper, based on the conception of ciphertext policy attribute-based encryption (CP-ABE), a PIR scheme based on CP-ABE is proposed for preserving the personal privacy in LBS. Personal privacy in LBS is a topic worth studying. However, there are some issues to consider:
1. In the “Introduction”, the authors described that “the superiority of this scheme can be reflected in both privacy preservation and quality of service”. However, the quality of service is not represented in the scheme, if the quality of service is the innovation point, it should be described in detail in the scheme, and it needs to be reflected in the abstract and contribution.
2. The “3.1 The conception of CP-ABE based PIR” should describ in the “Preliminaries”, and the system architecture and privacy threats are more appropriate in the “CP-ABE based PIR”, the front of the scheme description.
3. What is the relationship between this scheme and literature 8, and what are the advantages of this scheme compared with literature 8?

Response to reviewer's comments

**Reviewer #1:** In this paper, the authors try to propose a CP-ABE based PIR by utilizing CP-ABE technology.
However, the following questions should be considered.
(1) Inadequate motivation and problem formulation: The introduction fails to convincingly motivate the need for a CP-ABE based PIR. The specific research problems and challenges that the paper aims to address are not clearly articulated. The claimed contributions are not well-positioned against prior works, making it hard to gauge their significance. A stronger case needs to be made for why the proposed CP-ABE based PIR is an important advancement over existing solutions.

We add the formulation for the threat model to further promote the innovation of the motivation, and add the proof for why the LPPCAP utilizes CP-ABE based PIR to preserve the privacy of user in LBS. In addition, in the introduction, we also add the motivation of why we utilize CP-ABE based PIR and the claim the contribution of this work.
(2) Lack of technical depth and insights: The proposed construction does not offer significant technical innovations beyond combining existing techniques from CP-ABE based PIR. The paper lacks new concepts, insights, or design principles that push forward the state-of-the-art in this domain.

We change the construction of this manuscript and emphasize the innovation of LPPCAP to further demonstrate the insights of this manuscript.
(3) In this paper, the authors proposed a CP-ABE based PIR by utilizing CP-ABE technology. However, the authors do not give the related works about CP-ABE.

We add some CP-ABE and PIR algorithms used in LBS in the related work to demonstrate these schemes can be used in LBS. However the quality of service of these existing schemes is weaker than LPPCAP and we demonstrate this in the experiment result.
(4) The authors proposed a CP-ABE based PIR. However, they do not give the concrete name of the related algorithms.

We change the proposed scheme into LPPCAP and then modify the whole manuscript.
(5) The proposed CP-ABE based PIR is not complete. Especially, key generation algorithm is not given. How can you encrypt and decrypt?

We add the key generation algorithm in 3.2.

**Reviewer #2:**

1. It is not clear, what is the role of f(x) in algorithm 1?

We delete the f(x) in algorithm 1.

2. No threat model is presented in the paper. It will be good to include the threat model and present the security analysis accordingly.

We add the threat model in 2.1, and formulate it with probability$p(id\to l,q|b)$ and then present the security analysis in 4.1.

3. It is not clear whether the LBS will know who is sending the query or not? If the LBS knows the individual who sends the query then privacy is lost.

We add the process of how the LBS server know the query and location of the user in the threat model in 2.1, in this part, the way of privacy leakage of user is given.

4. Decryption proof of correctness may be with all steps involved. It is not clear how the second step is derived from the first step.

We add the precise step in algorithm 4, so as to give a clear steps for decryption.
5. Zero Knowledge conceal is used in table 2 however no where it is described.

We add the description of Zero Knowledge leakage about our scheme below table 2.

6. Figure 8 should be rechecked. X-axis is not clear. Is it time or the number of queries? Similarly figure 6.

The X-axis of figure 8 is queries, and we change all figures in the whole manuscript.

7. The novelty should be clearly presented.

We change the introduction as well as the abstract to further emphasize the novelty of this manuscript.

**Reviewer #3:** In this manuscript, in this paper, based on the conception of ciphertext policy attribute-based encryption (CP-ABE), a PIR scheme based on CP-ABE is proposed for preserving the personal privacy in LBS. Personal privacy in LBS is a topic worth studying. However, there are some issues to consider:

1. In the “Introduction”, the authors described that “the superiority of this scheme can be reflected in both privacy preservation and quality of service”. However, the quality of service is not represented in the scheme, if the quality of service is the innovation point, it should be described in detail in the scheme, and it needs to be reflected in the abstract and contribution.
The quality of service of LPPCAP is a part of innovation, so we add the reflection of this point in both the abstract and contribution.
2. The “3.1 The conception of CP-ABE based PIR” should describ in the “Preliminaries”, and the system architecture and privacy threats are more appropriate in the “CP-ABE based PIR”, the front of the scheme description.

We change the conception of LPPCAP in the “Preliminaries”, and then change the system architecture and the threat model to further appropriate to the conception of LPPCAP in 2.3.
3. What is the relationship between this scheme and literature 8, and what are the advantages of this scheme compared with literature 8?

The basic idea and conception of our proposed LPPCAP is derived from literature 8, however, in literature 8, the proposed scheme is mainly in theoretical and without any practice. So in this manuscript,we attempt to utilize the theoretical result in LBS to further the utilization of protocol proposed in literature. In addition, LPPCAP can be seen as a practical of the theory produced in literature [8]. We add the description of above in 4.1.
