# Supplementary material for: A location-based service scheme with attribute information privacy
Source: PLoS One. 2024 Sep 6;19(9):e0309919. doi: 10.1371/journal.pone.0309919 (PMC11379283; doi:10.1371/journal.pone.0309919)
Supplement: Attachment — Submitted filename: Response to Reviewers.docx [file pone.0309919.s003.docx]

Reviewers' comments:
**Reviewer #2:** The grammatical error in the revision part (in yellow color) has to be rechecked. The sentences are not complete or not conveying the meaning.

**Reviewer #4:** In this scheme, query and feedback are encrypted with security two-parties calculation by the user and the LBS server, so as not to violate any personal privacy and decrease the processing time in encrypting the retrieved information. In addition, this scheme can also preserve the attribute privacy of users such as the query frequency as well as the moving manner. This article is well written, I think it can be accepted, while the following comments should be revised.
(1) The title of the picture is recommended to be placed below the picture, not above it. (2) In the figure of experimental results, it is suggested to add references for comparison schemes. (3) A related work, fct: a fully-distributed context-aware trust model for location based service recommendation, is suggested to be discussed or compared in related work. (4) It is recommended that the serial number before Introduction be changed to 1.

**Response to reviewer's comments**

**Reviewer #2:** The grammatical error in the revision part (in yellow color) has to be rechecked. The sentences are not complete or not conveying the meaning.

We rechecked the revision part (in yellow color) and changed these sentences.

**Reviewer #4:** In this scheme, query and feedback are encrypted with security two-parties calculation by the user and the LBS server, so as not to violate any personal privacy and decrease the processing time in encrypting the retrieved information. In addition, this scheme can also preserve the attribute privacy of users such as the query frequency as well as the moving manner. This article is well written, I think it can be accepted, while the following comments should be revised.
(1) The title of the picture is recommended to be placed below the picture, not above it. (2) In the figure of experimental results, it is suggested to add references for comparison schemes. (3) A related work, fct: a fully-distributed context-aware trust model for location based service recommendation, is suggested to be discussed or compared in related work. (4) It is recommended that the serial number before Introduction be changed to 1.

(1) We change the title of the picture to the place below the picture.
(2) The references are added in figure of experimental results.
(3) A related work is discussed in related work, and we added it as “Liu et al. [30] also proposed a fully-distributed context-aware trust model for location based service. ” in section 2.
(4) The serial number before Introduction is changed to 1.
